# The Effects of Low-Dose Ketamine on Acute Pain in an Emergency Setting: A Systematic Review and Meta-Analysis

**DOI:** 10.1371/journal.pone.0165461

**Published:** 2016-10-27

**Authors:** Eun Nam Lee, Jae Hoon Lee

**Affiliations:** 1 Department of Nursing, Dong-A University, Daesin Gonwon-Ro, Seo-Gu, Busan, South Korea; 2 Department of Emergency Medicine, Dong-A university College of Medicine, Daesin Gonwon-Ro, Seo-Gu, Busan, South Korea; Nanjing University Medical School Affiliated Nanjing Drum Tower Hospital, CHINA

## Abstract

**Objectives:**

Currently ketamine is not used often as an analgesic in the emergency department (ED). Nonetheless, it can increase the efficiency of opioids and decrease their side effects. The purpose of this systematic review and meta-analysis was to evaluate whether low-dose ketamine in the ED provides better analgesia with fewer adverse effects.

**Methods:**

The PubMed, EMBASE, and Cochrane Library databases were searched by two reviewers independently (last search performed on January 2016). Data were also extracted independently.

**Results:**

A total of 6 trials involving 438 patients were included in the current analysis. Our subgroup analysis of pain reduction indicates that the favorable effects of ketamine were similar or superior to those of placebo or opioids, although these effects were heterogeneous. However, low-dose ketamine was associated with a higher risk of neurological (relative risk [RR] = 2.17, 95% confidence interval [CI] = 1.37–3.42, P < 0.001) and psychological events (RR = 13.86, 95% CI = 4.85–39.58, P < 0.001). In contrast, the opioid group had a higher risk of major cardiopulmonary events (RR = 0.22, 95% CI = 0.05–1.01, P = 0.05).

**Conclusions:**

The efficiency of ketamine varies depending on the pain site, but low-dose ketamine may be a key agent for pain control in the ED, as it has no side effects. It may also help to reduce the side effects of opioids.

## Introduction

Acute pain management is an important issue in the context of symptomatic care of patients in the emergency department (ED). Recently, opioids such as hydromorphine and remifentanil have been used to treat patients with acute pain. Indeed, opioids are the mainstay of acute pain management, along with non-steroidal anti-inflammatory drugs, acetaminophen, gabapentin, and regional treatments. Nonetheless, it is not desirable to administer opioids repeatedly to patients with acute pain, as they may lead to serious adverse effects such as hypotension and hypoxemia [1].

Ketamine is an N-methyl-D-aspartate (NMDA) receptor antagonist. It induces the synthesis and release of nitric oxide [2], and binds to μ receptors to increase the effectiveness of opioid-induced signaling [3]. In addition, recent studies have found that ketamine has anti-inflammatory effects [4–5]. Taken together, these actions of ketamine appear to contribute to analgesia against acute pain.

The efficacy of ketamine as an NMDA antagonist in the absence of opioids has been confirmed in many studies. Nonetheless, in the ED, ketamine is rarely used as an analgesic. Only one systematic review has evaluated the effects of a low-dose ketamine bolus infusion in patients visiting the ED. However, the results of this study were inconclusive and difficult to apply clinically. The systematic review included four randomized controlled trials (RCTs), but it also included two articles that used old data and had heterogeneous populations [6].

Ketamine at doses greater 1 mg/kg has commonly been used as a dissociative anesthetic either to induce anesthesia, or for brief procedures in a perioperative setting. Low-dose ketamine, which is defined as a bolus dose of less than 0.3 mg/kg, may also be used to control perioperative, pediatric, chronic, or cancer-related pain as adjuvant analgesia. To date, many systemic reviews have addressed these uses of ketamine [7–10]. Ketamine at doses of 0.5–1 mg/kg represents a gray zone and has variable effects on different patients. These borderline doses of ketamine cause many side effects, including neuropsychiatric problems [11,12], such as short-term hallucinations [13,14], unpleasant dreams, or acute psychosis [15]. Moreover, when 1–3 mg/kg of ketamine is administered, more than one-third of the patients may experience these symptoms [16]. Researchers have not clearly found the underlying reasons for neuropsychiatric side effects with low-dose ketamine use.

The aim of this review is to determine whether the analgesic effects of low-dose (0.3 mg/kg or less) ketamine delivered by intravenous bolus for the treatment of patients in the ED, are similar to those of opioids. In order to assess analgesic effects, a visual analogue scale (VAS) or a numeric rating scale (NRS) were used. We also determined whether the neuropsychiatric adverse effects of ketamine can be tolerable. We wished to know whether ketamine could be used as primary management for acute pain. Thus, the primary outcome in our study was pain reduction and the secondary outcome was a decrease in the occurrence of adverse effects.

## Methods

### Registration Number

We have registered the protocol for this systematic review in PROSPERO, which is the international prospective register of systemic reviews. (available from http://www.crd.york.ac.uk/PROSPERO/display_record.asp?ID=CRD42015024337, S1 Text).

### Data Sources and Search Strategy

To identify randomized controlled trials (RCT)s testing the effects of intravenous ketamine injection on acute pain in the ED, we searched MEDLINE, EMBASE, and the Cochrane Central Register of Controlled Trials (CENTRAL) for studies published between 1962 and January 2016. The search terms combined “ketamine and acute pain”, “ketamine and pain management”, “ketamine or acute pain or pain management”, and “randomized controlled trials” as detailed in the appendix. In addition, when we selected a trial, we reviewed its bibliography for additional unidentified studies. Two researchers conducted the literature search independently and reviewed the study title and abstract for every retrieved reference. They removed duplicates that were identified in multiple database searches.

### Study Selection

We identified whether studies met the inclusion criteria first on the basis of the title, then based on the abstract, and finally based on the full paper. The inclusion criteria were as follows: (1) only randomized controlled trials were included because retrospective and prospective studies are not useful for assessing the risk of bias; (2) studies with parallel group designs comparing ketamine with a placebo, morphine, or fentanyl for the treatment of acute pain in adults were included because independent use of ketamine and opioids or placebo was needed in each study to ascertain the effects of ketamine; (3) subjects had to have moderate to severe pain (a score of ≥5 out of 10 on the NRS or more than a half score on the VAS) so that they could be treated with ketamine; (4) to compare the doses and actions of ketamine to those of opioids, we only included studies that used a low-dose of intravenous ketamine to reduce side effects. Many studies were excluded based on the following criteria: (1) studies that investigated the effects of ketamine on perioperative pain such as subacute pain after anesthesia, cancer-related pain or other non-acute pain; (2) those that induced experimental pain for research purposes; (3) those that included non-intravenous administration or ketamine or a mixed administration of drugs (one of three prospective studies we searched for simultaneously used ketamine and hydromorphine without a control group and another was used intranasal ketamine); and (4) studies using children who were younger than 14 years old, as adult doses of intravenous ketamine have even greater effects.

The primary outcome measure was the acute pain score 30 minutes after the injection of ketamine, placebo, or opioids. The secondary outcome measures were the cumulative frequencies of all the adverse events described in the studies (*e*.*g*. gastrointestinal, neurological, psychological, and cardiopulmonary adverse effects).

Two researchers screened the databases independently and retrieved potentially relevant studies. Another two reviewers assessed the eligibility of the full text of the articles. They resolved discrepancies by discussion when necessary.

### Data Extraction and Quality Assessment

Data from the eligible articles were extracted according to pre-determined criteria by two reviewers. We extracted data regarding mean age, dose of ketamine and opioids, degree of pain reduction after 30 minutes, and side effects. If the article did not report the pain score at 30 minutes after injection, we extracted the value that was measured closest to that time. We also extracted the change in the pain intensity score between baseline and after treatment or the absolute post-treatment score. We hypothesized that VAS and NRS were strongly correlated and could be used interchangeably for the assessment of patients with acute pain in the ED, although this is controversial [17,18].

Two reviewers independently assessed and discussed the risk of bias in each study based on the recommendations provided in the Cochrane Handbook for Systematic Reviews of Interventions, version 5.1.0 (http://www.handbook.cochrane.org). In their appraisal, the reviewers considered random sequence generation, allocation concealment, missing or incomplete outcome data, selective reporting and blinding of patients, study personnel, and outcome assessors.

### Data Synthesis and Analysis

Data were analyzed using the Cochrane Collaboration‘s software (Review Manager [RevMan] Version 5.3, Copenhagen: The Nordic Cochrane Centre). Heterogeneity among the studies was evaluated using the I^2^ and chi-squared tests. If the heterogeneity test revealed a statistical significance, a random effects model was used (I^2^ < 50%; P > 0.1) because of unexplained heterogeneity in the subgroup analysis. We utilized standardized mean differences (SMD) and 95% confidence intervals (CI). The efficacy of ketamine was compared with that of opioids in terms of adverse effects; the odds ratio and 95% CIs were used for this purpose. All reported P-values are two-sided.

## Results

### Study Selection

**Fig 1:**
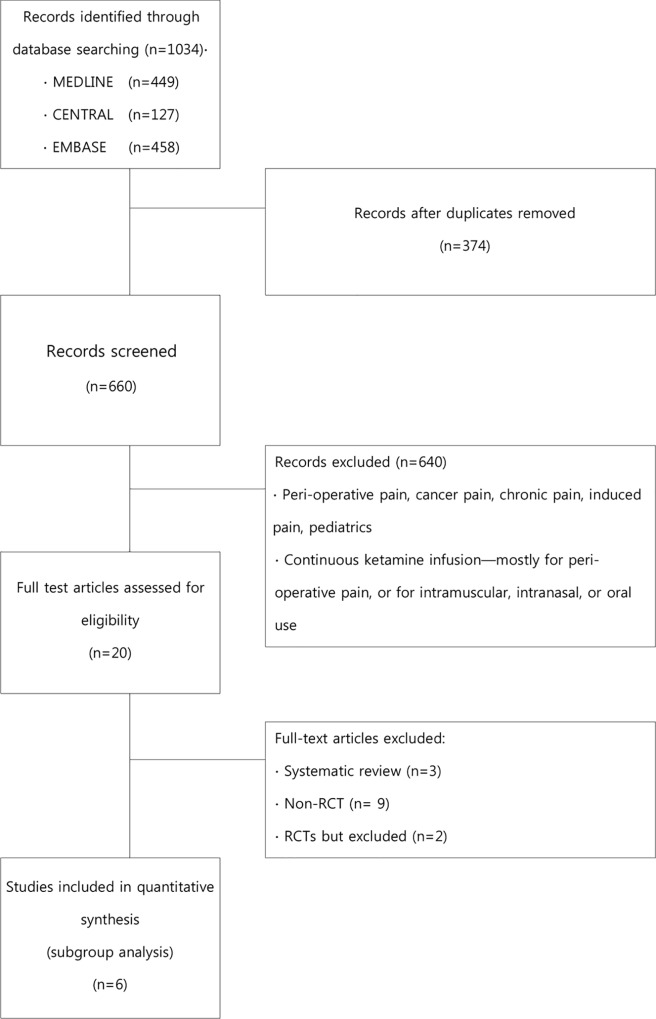
Study Selection PRISMA Flowchar.

Our searches identified 1,034 potentially relevant studies. However, 1,014 of these studies were excluded (Fig 1). Among the eligible 20 studies, 3 systematic reviews, 9 non-RCTs [19–27] and 2 RCTs [28,29] were excluded: a case series and a letter [19,26], three retrospective studies [23–25], studies that used treatments other than intravenous infusion [20,21,23], a prospective study in which both ketamine and hydromorphine were simultaneously used without a control group [22], and another study that was a prospective cohort study with multiple biases, such as selection, performance, detection, and reporting bias [27]. None of the above excluded studies were RCTs. Two RCTs also were exempted: one where ketamine was continuously infused in a perioperative setting [29], and another that assessed pain 6 months after ketamine injection (long-term pain prevalence) [28]. The remaining 6 studies [1,30–34] that fulfilled the inclusion criteria form the basis of this review. The key data from six RCTs included are summarized in Table 1.

**Table 1:** Characteristics of the Included Studies.

Studies	Sample size	Ages (mean)	Intervention	Control	Pain reduction	Side effects
Galinski et al. (2006)	65 patients	18–70 years (mean 35–40)	Ketamine 0.2 mg/kg	Placebo	VAS at 30 minutes	• Nausea/vomiting • Neuropsychological events • Itching • Bradypnea
Messengers et al. (2008)	63 patients	14–65 years (mean 35.6–43.2)	Ketamine 0.3 mg/kg	Fentanyl 1.5 μg/kg	NRS during procedure	• Cardiorespiratory clinical events
Jennings et al. (2012)	135 patients	Over 18 years (mean 41–45)	Ketamine 10–20 mg followed by 10 mg every 3 minutes	Morphine 5 mg followed by 1–5 mg every 5 minutes	NRS at 20 minutes	• Nausea/vomiting • Decreased consciousness • Nystagmus/visual disturbance • Disorientation • Emergence phenomenon • Hypertension • Tachycardia • Enhanced skeletal tone
Miller et al. (2014)	45 patients	18–59 years (mean 29–31)	Ketamine 0.3 mg/kg followed by the same dose	Morphine 0.1 mg/kg followed by the same dose	NRS at 20 minutes	• Nausea/vomiting • Dysphoria • Hallucination • Dizziness • Lightheadedness • Drowsiness • Numbness • Pruritus • Decreased oxygen saturation
Beaudoin et al. (2014)	40 patients	18–65 years (mean 32.5–37.5)	Ketamine 0.3 mg/kg	Placebo	NRS at 30 minutes	• Dizziness • Lightheadedness • Nausea/vomiting • Dysphoria/confusion • Hypotension • Respiratory depression • Sinus tachycardia
Motov et al. (2015)	90 patients	18–55 years (mean 35–36)	Ketamine 0.3 mg/kg	Morphine 0.1 mg/kg	NRS at 30 minutes	• Nausea • Dizziness • Disorientation • Mood changes

VAS, visual analogue scale; NRS, numeric rating scale.

### Study Quality

According to the Cochrane Collaboration‘s Tool for Assessing the Risk of Bias, three of the six studies had a low risk of bias in all six categories. All studies reported the details of allocation concealment. Although one study described only the sequence generation process as randomized, there was no explanation of this term [32].

**Fig 2:**
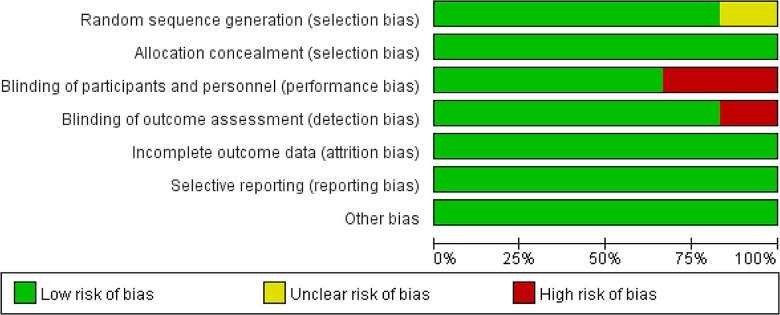
Risk of Bias Graph.

**Fig 3:**
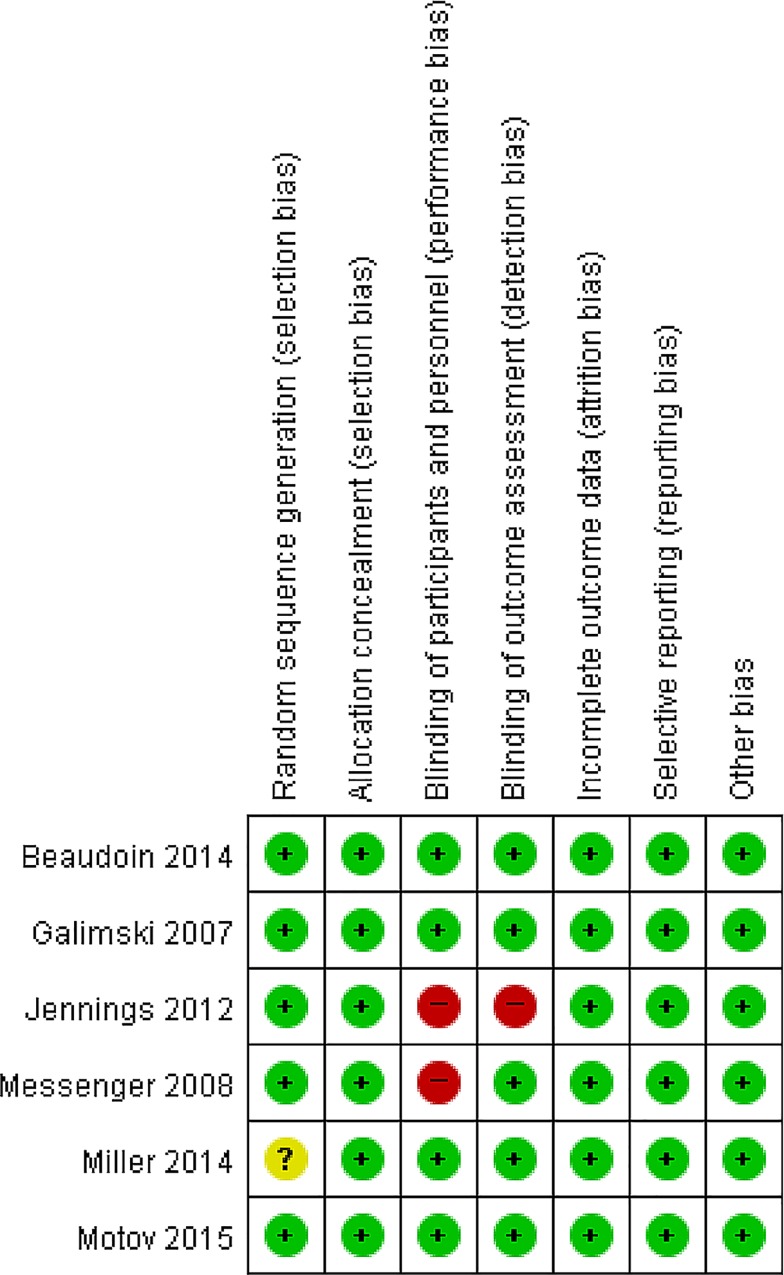
Risk of Bias Summary.

Two of the RCTs, those of Messenger et al. and Jennings et al. reported evidence of compromised blinding [1,33]. Specifically, the physician had to observe the subjects’ ocular response during sedation by ketamine, and the nystagmus or muscle twitching that result from ketamine administration may weaken blinding. In the Jennings trial, after an initial bolus, a second analgesia was administered by the treating paramedics at a dose of 1–5 mL every 5 minutes in the morphine arm and at a dose of 1mL every 3 minutes in the ketamine arm. In addition, the verbal NRS was assessed and recorded by the treating paramedic. Therefore, assessor blinding may have been compromised. The risks of bias assessment for all included studies are summarized in Figs 2 and 3.

### Descriptions of the RCTs Included in the Analysis

In all studies, ketamine was administered as an intravenous bolus. Four studies used ketamine at a dose of 0.3 mg/kg [1,30,32,34], while the remaining studies used initial ketamine doses of 0.2 mg/kg [31] or 10–20 mg [33]. Ketamine was compared to a saline placebo in two trials [31,34], to 0.1 mg/kg morphine [30,32] or 5 mg morphine [33] in three trials, and to 1.5 μg/kg fentanyl in one trial [1].

Three trials pre-specified rescue analgesia using morphine or fentanyl if necessary [30,31,34]. In one trial, repetitive injections were administrated to treat pain with no established rescue analgesia [33], while propofol was needed for more adequate sedation in another trial [1]. In the remaining trial, the provider conferred with the patient regarding when additional analgesia was provided [32].

We could have conducted a meta-analysis of the reductions in pain scores in our six RCTs. Such an analysis would have included 438 subjects who had visited the ED with acute pain. However, we did not perform this analysis because each study had a different control group. Instead, we carried out a subgroup-analysis of pain reduction. We did however perform a meta-analysis of the adverse effects, wherein there was no heterogeneity.

### Primary Outcome: Change in Pain Intensity

**Fig 4:**
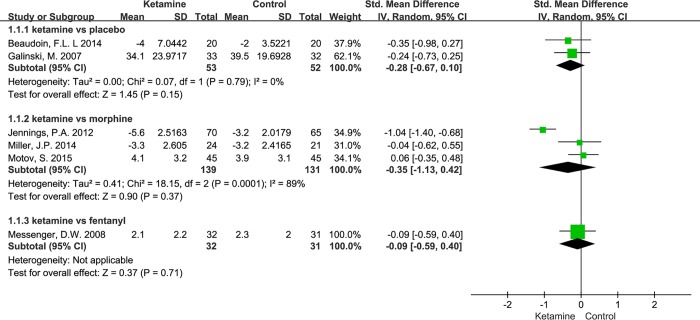
Subgroup Analysis of Pain Reduction.

In five trials, researchers used the NRS as a pain scale, whereas one trial used the VAS [31]. In all trials, pain intensity was assessed upon arrival at the ED, as well as 20 or 30 minutes after ketamine or opioid injection. Subgroup analysis indicates that ketamine leads to a trends for reduced pain scores, although this is not homogeneously in the morphine group (heterogeneity: chi^2^ = 18.15, I^2^ = 89%, P < 0.001; Fig 4).

In the two studies where ketamine was compared to a placebo and fentanyl, respectively, rescue analgesia was used less in the ketamine group, although the pain scores did not differ significantly [1,31]. In another study, 0.3 mg/kg ketamine appeared to significantly reduce the need for rescue analgesia (P < 0.04). However, the effects of 0.15 mg/kg ketamine were not significantly different than those of the placebo (P < 0.1) [34]. Specifically, the median doses of rescue analgesia in the placebo, 0.15 mg/kg ketamine, and 0.3 mg/kg ketamine arms were 6.1 mg, 5.4 mg, and 4.3 mg, respectively. When ketamine was compared to opioids, its effects on analgesia and the reduction of rescue analgesia were similar to those of opioids [30].

### Secondary Outcome: the Incidence of Adverse Effects

We categorized adverse effects into gastrointestinal (nausea and vomiting), neurological (dizziness, headache, light-headedness, nystagmus, visual disturbance, drowsiness, numbness, or increased skeletal tone), psychological (hallucination, dysphoria or confusion, agitation, disorientation, or mood change) and cardiopulmonary (major: hypoxia and hypotension; minor: tachycardia and hypertension).

**Fig 5:**
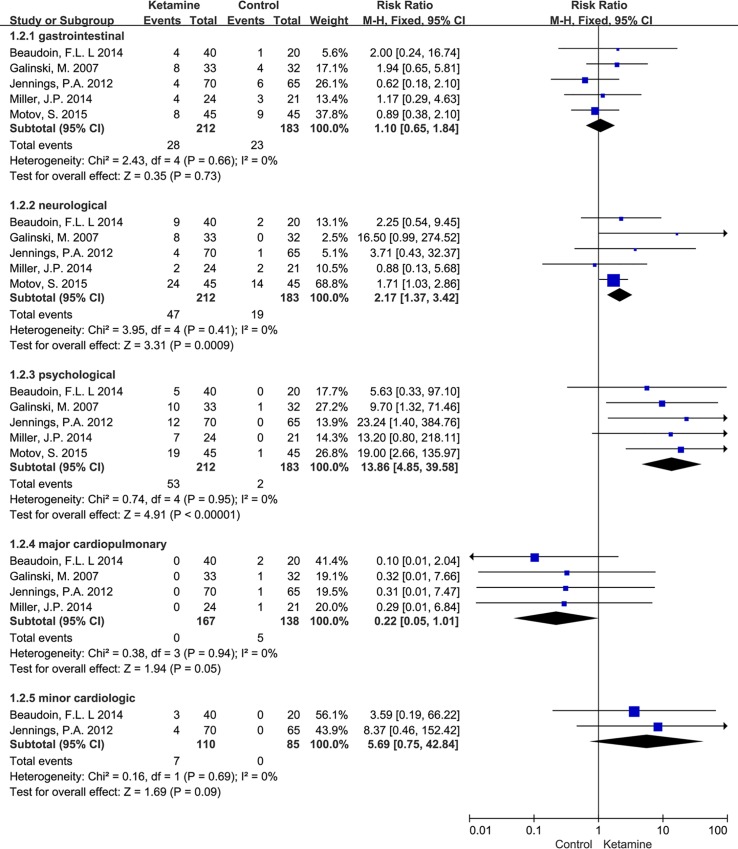
Meta-analysis of the Incidence of Adverse Events.

Compared to the placebo and opioid groups, the ketamine group was associated with a greater risk of neurological (RR = 2.17, 95% CI = 1.37–3.42, P < 0.001), psychological (RR = 13.86, 95% CI = 4.85–39.58, P < 0.001) and minor cardiologic events (RR = 5.69, 95% CI = 0.75–42.84, P = 0.09). On the other hand, there was a higher risk for major cardiopulmonary events in the opioid group and the placebo plus rescue opioid group. Major cardiopulmonary events were not observed in the ketamine group (RR = 0.22, 95% CI = 0.05–1.01, P = 0.05). There were no differences in the incidence of gastrointestinal adverse events (RR = 1.1, 95% CI = 0.65–1.84, P = 0.73; Fig 5).

## Discussion

Heterogeneity among the studies was significant because we included only small studies that focused on ketamine. This is a limitation when systemically reviewing this topic. We attempted to decrease heterogeneity by limiting the review to RCTs that only used intravenous ketamine in adults.

Despite this heterogeneity, our analysis suggests that intravenous ketamine for pain reduction and opioid sparing has equivalent or better effect when compared to opioids or placebo. However no significant differences were observed in either regard, other than in a study by Jennings et al. In addition, definite pain reduction should be observed in the ketamine group when compared to a placebo group. However, this was not the case, as seen in Fig 4. This may be due to the fact that the effect of intravenous ketamine can be strongly influenced by the site of pain. Our studies compared ketamine to placebo in an eligible population presenting with trauma and severe acute pain [31]. The study population had no specific diseases such as cardiac, renal, or cerebral diseases [34] and was very diverse. Most recently, the effect of intravenous ketamine on the site of pain in a perioperative setting was investigated in a review article. This article demonstrated that ketamine is effective in various surgeries, although there were some studies that were controversial [35]. Furthermore, a systemic review of perioperative intravenous ketamine reported analgesic benefits in painful procedures, including upper abdominal, thoracic, and major orthopedic surgeries [7]. The different effects of ketamine on different pain sites and the exact underlying mechanisms have not yet been completely described.

Generally, the amount of rescue opioid that is administered is smaller in the ketamine groups [31]. It is likely that the measured effects of ketamine contribute to this. However, most patients in the ketamine group in our studies required repetitive rescue opioids as often or more frequently than in the morphine group to control severe pain [30,32,34]. Similarly, the reason for this observation may be that small doses of ketamine were used, or that about 70% of the studies included patients with abdominal pain, where ketamine had a lower probability of having an effect. This is especially true for the lower abdomen.

The combination of low-dose ketamine and morphine reduces morphine requirements by approximately 26%–60% in patients with moderate to severe acute pain [11,31,36,37]. Johansson et al report that only half as much rescue morphine was administered to patients receiving 0.2 mg/kg ketamine when compared to control group, which used morphine [27].

The patients included in our six RCT studies had scores of more than 50% on the NRS or the VAS. Ketamine should be used in the ED to rescue patients who continue to have severe pain despite routine treatment. Mildly painful procedures and controllable pain do not benefit from ketamine. For instance, in surgery associated with mild pain (VAS equivalent: <40% of the maximum score on any pain scale) ketamine is not beneficial [7].

Opioids inhibit nociception by activating the μ receptor, predominantly at presynaptic sites [38], They also activate monoaminergic descending pathways at the spinal level. However, they also activate NMDA receptors, and result in postsynaptic neuronal hyper-excitability of pain (central sensitization). This causes hyperalgesia. In addition, the patient will develop a tolerance to opioids, which leads to chronic pain [10,39,40].

In contrast to opioid, ketamine binds spinal μ receptors and increases the effectiveness of opioid-induced signaling [3]. In addition, ketamine functions as an NMDA antagonist and preferentially acts postsynaptically, leading to a reduction in hyperexcitability [41]. Therefore, ketamine blockage of NMDAs may improve the efficacy of opioids and have an opioid-sparing effect. Thus, ketamine not only prevents the serious adverse effects of opioids, but also inhibits the chronic pain that develops as a result of opioid tolerance.

Low-dose ketamine inhibits nociception through its high affinity for the NMDA receptor [42,43]. It may be that low-dose ketamine interacts more selectively with NMDA receptors, whereas, at full-anesthetic doses, ketamine activates different types of opioid receptors with various affinities (μ, κ, and σ opioid receptors) [44,45].

High-dose ketamine is also less desirable because it causes hallucinations, nightmares, nausea, dizziness, and blurred vision, among other adverse effects. In addition, there is insufficient evidence regarding the amount of ketamine that should be administered in the ED to optimize efficacy and minimize side-effects. Studies have reported that in postoperative settings other than those after gynecological or bowel surgery, 2–3 μg/kg/minute of ketamine does not lead to increased side effects. Similarly, a 5 mg patient-controlled, intravenous bolus of ketamine does not increase side effects in patients with cancer [12,46,47]. Nonetheless, no investigations have yet determined the amount of ketamine that can be used in patients with severe acute pain in the ED. In the studies that we reviewed, a 1.5–3 mg/kg intravenous bolus of ketamine was defined as a low dose. Nonetheless, even such a small dose had neuropsychiatric side effects that may preclude the routine use of this drug.

In the RCTs we selected, administration of ketamine at doses of only 0.15–0.3 mg/kg had considerable neuropsychiatric side effects. To ensure that ketamine is used safely without neuropsychiatric side effects, a very low-concentration intermittent intravenous bolus or a 2–3 μg/kg/minute continuous infusion, might be considered. This may prevent the overuse of opioids while effectively treating severe acute pain in the ED.

Use of only opioids to treat acute pain causes shallow breathing and cough. This leads to inadequate ventilation in the patients, which may cause decreased saturation [11]. Furthermore, adverse events such as nausea, hypoxia, and hypotension are associated with opioid use. In addition, wakefulness is significantly lower with opioid use than when ketamine is used as an adjuvant [1,11,47]. In our review, major cardiovascular events appeared more frequently as a side effect with the use of opioids.

Further studies should be carried out regarding the effects of ketamine in the ED in the future. The effects of ketamine in specific diseases or pain sites should be studied, as different doses of ketamine and specific pain sites may affect pain reduction, opioid sparing, and adverse effects. In addition, continuous infusion of ketamine is appropriate to use in the ED. In many studies that used perioperative intravenous ketamine, continuous ketamine infusion at a dosage of 2 μg/kg/minute during the operation, or infusion for 24 hours, reduced the amount of morphine necessary with no significant side effects [46,48–50]. Research on early applications of continuous ketamine infusion, as opposed to intermittent use, is needed in patients with severe pain who present in the ED.

There were some limitations to our systematic review. The meta-analysis was not performed because it failed to analyze the overall differences in pain reduction with ketamine vs. control in the 6 studies included. The control groups were not categorized into placebo, morphine, and fentanyl groups. Moreover, there was substantial statistical heterogeneity between the subgroups when pain scores were compared between the ketamine and morphine groups. This likely occurred because of differences in the pain values recorded in and extracted from each study. The pain scores were either the absolute values measured after intervention or presented as changes from baseline. These scores were analyzed within subgroups. Moreover, we could not perform a sensitivity analysis and meta-regression to check heterogeneity because we only included a small number of studies, which were themselves divided into subgroups. For the same reason, we did not assess the risk of publication bias. Finally, the numbers of patients and the studies in each comparison were small, which greatly restricted the quality of the evidence.

In conclusion, our data support the routine use of ketamine for the treatment of severe pain (>5 NRS) in the ED as a first treatment, as it was equivalent to opioids when compared to morphine/fentanyl in all trials studied. In addition, it leads to a trend towards increased analgesia in trials compared to placebo. There is also a trend towards fewer cardiopulmonary side effects with ketamine use.

## Supporting Information

S1 Checklist PRISMA Checklist for the Systematic Review of Ketamine. (DOC)

S1 Text CRD of the Systematic Review of Ketamine. (PDF)
