# Supplementary material for: The Effects of Low-Dose Ketamine on Acute Pain in an Emergency Setting: A Systematic Review and Meta-Analysis
Source: PLoS One. 2016 Oct 27;11(10):e0165461. doi: 10.1371/journal.pone.0165461 (PMC5082866; doi:10.1371/journal.pone.0165461)
Supplement: S1 Text — (PDF) [file pone.0165461.s002.pdf]

PROSPERO International prospective register of systematic reviews

Review title and timescale

1 Review title

Give the working title of the review. This must be in English. Ideally it should state succinctly the interventions or exposures being reviewed and the associated health or social problem being addressed in the review.

The effect of intravenous ketamine for patients with acute pain in emergency setting

2 Original language title

For reviews in languages other than English, this field should be used to enter the title in the language of the review. This will be displayed together with the English language title.

3 Anticipated or actual start date

Give the date when the systematic review commenced, or is expected to commence.

01/07/2015

4 Anticipated completion date

Give the date by which the review is expected to be completed.

27/11/2015

5 Stage of review at time of this submission

Indicate the stage of progress of the review by ticking the relevant boxes. Reviews that have progressed beyond the point of completing data extraction at the time of initial registration are not eligible for inclusion in PROSPERO. This field should be updated when any amendments are made to a published record.

The review has not yet started ×

Review stage	Started	Completed
Preliminary searches	Yes	No
Piloting of the study selection process	No	No
Formal screening of search results against eligibility criteria	No	No
Data extraction	No	No
Risk of bias (quality) assessment	No	No
Data analysis	No	No

Provide any other relevant information about the stage of the review here.

PRISMA was not yet completed.

Review team details

6 Named contact

The named contact acts as the guarantor for the accuracy of the information presented in the register record.

Jae Hoon Lee

7 Named contact email

Enter the electronic mail address of the named contact.

leetoloc@dau.ac.kr

8 Named contact address

Enter the full postal address for the named contact.

Emergency department, Dong-A University Medical Center, 26 DaeSingGongWon-Ro, Busan, Korea, 602-812

9 Named contact phone number

Enter the telephone number for the named contact, including international dialing code.

82-10-4847-7916

10 Organisational affiliation of the review

Full title of the organisational affiliations for this review, and website address if available. This field may be completed as 'None' if the review is not affiliated to any organisation.

Dong-A University Medical Center

Website address:
www.damc.or.kr

11 Review team members and their organisational affiliations
Give the title, first name and last name of all members of the team working directly on the review. Give the organisational affiliations of each member of the review team.

Title	First name	Last name	Affiliation
Professor	Jae Hoon	Lee	Dong-A University Medical Center
Professor	Eun Nam	Lee	Dong-A University
Mrs	Jin Hee	Kim	Dong-A University
Miss	Eun Hui	Choi	Dong-A University

12 Funding sources/sponsors
Give details of the individuals, organizations, groups or other legal entities who take responsibility for initiating, managing, sponsoring and/or financing the review. Any unique identification numbers assigned to the review by the individuals or bodies listed should be included.
This work was supported by the Dong-A University Research Fund.

13 Conflicts of interest
List any conditions that could lead to actual or perceived undue influence on judgements concerning the main topic investigated in the review.
Are there any actual or potential conflicts of interest?
None known

14 Collaborators
Give the name, affiliation and role of any individuals or organisations who are working on the review but who are not listed as review team members.

Title	First name	Last name	Organisation details

Review methods

15 Review question(s)
State the question(s) to be addressed / review objectives. Please complete a separate box for each question.
1. What are the best clinical management of acute pain which patients have in emergency setting?

2. What is the optimal dose that is combined ketamine and other opioid (morphine or fentanyl) at the time those work the best with low side effect (e.i. delirium, hallucination, nausea, vomitting..) and decrease a lot scores of acute pain?

3. Can ketamine be used for adult patients admited on emergency room as acute pain, not just that ketamine is applied for child or perioperative status or chronic pain like cancer?

4. Which is better management among intravenous ketamine + morphine, intravenous ketamine and intravenous morphine as management of acute pain for adult patients admitted on emergency setting?

16 Searches
Give details of the sources to be searched, and any restrictions (e.g. language or publication period). The full search strategy is not required, but may be supplied as a link or attachment.
The PubMed, EMBASE and the Cochrane Library databases will be searched as words like "acute pain" and "ketamine". English language publication period is 1995 ~ 2015.

17 URL to search strategy
If you have one, give the link to your search strategy here. Alternatively you can e-mail this to PROSPERO and we will store and link to it.

I give permission for this file to be made publicly available

Yes

18 Condition or domain being studied

Give a short description of the disease, condition or healthcare domain being studied. This could include health and wellbeing outcomes.

adult patients with acute pain in emergency setting. trauma pain, procedure pain, abdominal pain, etc. are examples for it. The outcomes could be presented as NRS or VAS score.

19 Participants/population

Give summary criteria for the participants or populations being studied by the review. The preferred format includes details of both inclusion and exclusion criteria.

inclusion criteria : adult patients with acute pain in emergency setting exclusion criteria : patients with the use of ketamine in child or perioperative status or chronic pain : patients with the oral or intranasal or subcutaneous use of ketamine without intravenous

20 Intervention(s), exposure(s)

Give full and clear descriptions of the nature of the interventions or the exposures to be reviewed

Ketamine, a noncompetitive N-methy-D-aspartate (NMDA)-receptor antagonist was shown to enhance opioid induced antinociception and besides, use combined ketamine and other opioids reduce side effect of ketamine and other opioid consumption. Ketamine is able to be used intranasal or oral or subcutaneous or intravenous. We will be gathering the data about only intravenous use of ketamine because many data by studies have been showed previously and painful patients have been frequently treated through the intravenous way in emergency department.

21 Comparator(s)/control

Where relevant, give details of the alternatives against which the main subject/topic of the review will be compared (e.g. another intervention or a non-exposed control group).

Use combined ketamine and other opioid is recommended recently because of side effect of ketamine. We will compare ketamine with other opioids that are used generally in emergency setting. Effect of low dose ketamine + morphine or midazolam or fentanyl was acknowledged from last years and the benefit of low dose ketamine was already announced when the patients are child or under the surgery or have chronic pain like cancer as several systemic review. But, study about ketamine applied for the adult patients with acute pain in emergency setting is not known well and there is a little controversy and low evidence. Therefore we are about to be studying about the use of ketamine in adult patients with acute pain in emergency setting.

22 Types of study to be included initially

Give details of the study designs to be included in the review. If there are no restrictions on the types of study design eligible for inclusion, this should be stated.

Randomized Controlled Study

23 Context

Give summary details of the setting and other relevant characteristics which help define the inclusion or exclusion criteria.

24 Primary outcome(s)

Give the most important outcomes.

VAS score, NRS score

Give information on timing and effect measures, as appropriate.

Information for pain scale from RCT papers

25 Secondary outcomes

List any additional outcomes that will be addressed. If there are no secondary outcomes enter None.

side effects (nausea, vomitting, dizziness, delirium, hallucination, sedation...)

Give information on timing and effect measures, as appropriate.

event number of side effects

26 Data extraction, (selection and coding)

Give the procedure for selecting studies for the review and extracting data, including the number of researchers involved and how discrepancies will be resolved. List the data to be extracted.

We will extract the following data from the included articles: title, author name, publication data, number of patients, study design (including grouping, blinding, etc.), description of intravenous ketamine's use (amounts of use), pain

score (NRS, VAS) and side effects.

27 Risk of bias (quality) assessment
State whether and how risk of bias will be assessed, how the quality of individual studies will be assessed, and whether and how this will influence the planned synthesis.
We will use the Cochrane Handbook to assess the risk of bias for all articles. The following information will evaluate : random sequence generation, allocation concealment, blinding, incomplete outcome data, selective reporting and other bias. Assessment and evaluation will are conducted independently by two reviewers. Other two reviewers will are consulted to reconcile any disagreements. Subgroup analysis will be referred for evaluation of the heterogeneity of the randomized trials.

28 Strategy for data synthesis
Give the planned general approach to be used, for example whether the data to be used will be aggregate or at the level of individual participants, and whether a quantitative or narrative (descriptive) synthesis is planned. Where appropriate a brief outline of analytic approach should be given.
We suppose all data of our study is collected from RCT papers in where mostly descriptive synthesis will be used. If we need concrete information, we have to ask authors their data. The data aggregated through heterogeneity test will be analysed by meta-analysis or sub group meta-analysis.

29 Analysis of subgroups or subsets
Give any planned exploration of subgroups or subsets within the review. ‘None planned’ is a valid response if no subgroup analyses are planned.
Main analysis is comparing morphine group with ketamine + morphine group, but if we have enough data about fentanyl group and midazolam group, we will analyse the sub-groups.

Review general information

30 Type of review
Select the type of review from the drop down list.
Intervention

31 Language
Select the language(s) in which the review is being written and will be made available, from the drop down list. Use the control key to select more than one language.
English

Will a summary/abstract be made available in English?

32 Country
Select the country in which the review is being carried out from the drop down list. For multi-national collaborations select all the countries involved. Use the control key to select more than one country.
South Korea

33 Other registration details
Give the name of any organisation where the systematic review title or protocol is registered together with any unique identification number assigned. If extracted data will be stored and made available through a repository such as the Systematic Review Data Repository (SRDR), details and a link should be included here.

34 Reference and/or URL for published protocol
Give the citation for the published protocol, if there is one.
Give the link to the published protocol, if there is one. This may be to an external site or to a protocol deposited with CRD in pdf format.

I give permission for this file to be made publicly available
Yes

35 Dissemination plans
Give brief details of plans for communicating essential messages from the review to the appropriate audiences.
We will submit to a journal of emergency medicine.

Do you intend to publish the review on completion?
Yes

36 Keywords
Give words or phrases that best describe the review. (One word per box, create a new box for each term)
ketamine

acute pain

37 Details of any existing review of the same topic by the same authors
Give details of earlier versions of the systematic review if an update of an existing review is being registered, including full bibliographic reference if possible.
There are many articles of systemic review that are similar to our study. But most reviews were related with perioperative pain. Those was exempted from our exclusive criteria. There was the most similar study when it is compared with ours. (ACADEMIC EMERGENCY MEDICINE 2015;22:251–257) But, That just included a pediatric study and old RCT papers (1998, 2007, 2008) though there were many RCT papers recently (2012~2015).

38 Current review status
Review status should be updated when the review is completed and when it is published.
Ongoing

39 Any additional information
Provide any further information the review team consider relevant to the registration of the review.

40 Details of final report/publication(s)
This field should be left empty until details of the completed review are available.
Give the full citation for the final report or publication of the systematic review.
Give the URL where available.
